# Association of the *MTHFR* A1298C Variant with Unexplained Severe Male Infertility

**DOI:** 10.1371/journal.pone.0034111

**Published:** 2012-03-23

**Authors:** Abdelmajid Eloualid, Omar Abidi, Majida Charif, Brahim El houate, Houda Benrahma, Noureddine Louanjli, Elbakkay Chadli, Maria Ajjemami, Abdelhamid Barakat, Anu Bashamboo, Ken McElreavey, Houria Rhaissi, Hassan Rouba

**Affiliations:** 1 Human Genetic Unit, Research Department, Pasteur Institute, Casablanca, Morocco; 2 Cytogenetic Unit, Pasteur Institute, Casablanca, Morocco; 3 In Vitro Fecondation Center, Casablanca, Morocco; 4 Human Developmental Genetics Unit, Pasteur Institute, Paris, France; 5 Faculty of Sciences Ben M'sik, Casablanca, Morocco; Universidad Europea de Madrid, Spain

## Abstract

The methylenetetrahydrofolate reductase (*MTHFR*) gene is one of the main regulatory enzymes involved in folate metabolism, DNA synthesis and remethylation reactions. The influence of MTHFR variants on male infertility is not completely understood. The objective of this study was to analyze the distribution of the *MTHFR* C677T and A1298C variants using PCR-Restriction Fragment Length Polymorphism (RFLP) in a case group consisting of 344 men with unexplained reduced sperm counts compared to 617 ancestry-matched fertile or normozoospermic controls. The Chi square test was used to analyze the genotype distributions of MTHFR polymorphisms. Our data indicated a lack of association of the C677T variant with infertility. However, the homozygous (C/C) A1298C polymorphism of the *MTHFR* gene was present at a statistically high significance in severe oligozoospermia group compared with controls (OR = 3.372, 95% confidence interval CI = 1.27–8.238; p = 0.01431). The genotype distribution of the A1298C variants showed significant deviation from the expected Hardy-Weinberg equilibrium, suggesting that purifying selection may be acting on the 1298CC genotype. Further studies are necessary to determine the influence of the environment, especially the consumption of diet folate on sperm counts of men with different *MTHFR* variants.

## Introduction

Infertility is estimated to affect 10–15% of couples, and roughly half of these cases are due to the male factor [1]. Spermatogenic failure is the most common form of male infertility; however, in most cases the etiology remains unknown. Genetic abnormalities are thought to account for 15%–30% of male factor infertility and these can include Y chromosome microdeletions, chromosomal aberrations and rare single-gene defects [2]–[5]. Deleterious gene polymorphisms in key genes involved in testicular function, in combination with environmental insults, may be responsible for the reduced sperm numbers and poor sperm quality that are observed in many infertile men.

A possible candidate for genetic susceptibility to spermatogenic failure is the gene 5,10-Methylenetrahydrofolate reductase (*MTHFR*). *MTHFR* is an important regulatory enzyme in folate and homocysteine metabolism, which is necessary for a number of key biological cellular mechanisms [6], [7]. This regulatory enzyme catalyzes the reduction of 5,10-methylenetetrahydrofolate to produce 5- methyltetrahydrofolate, which is the methyl donor for the remethylation of homocysteine to methionine. Subsequently, methionine provides the methyl group for the formation of S-adenosylmethionine, the methyl donor for DNA methylation [8]. Methylation anomalies of sperm DNA has been linked to male infertility [9]. Reduced enzymatic activity due to *MTHFR* polymorphisms is associated with hyperhomocysteinemia that is considered as a risk factor for many diseases, including infertility [10]. Moreover, the activity of *MTHFR* is much higher in testis than in other major organs in the adult mouse, suggesting that it might play an important role in testicular function [11]. Two single nucleotide polymorphisms (SNPs) in the *MTHFR* gene, C677T (A→V) [12] and A1298C (E→A) [13], [14], are individually associated with a reduction in the biochemical activity of the enzyme. The *MTHFR* C677T variant decreases *MTHFR* activity and increases the homocysteine level. Similarly the *MTHFR* A1298C variant also reduces enzyme activity but to a lesser degree than C677T [12]–[15]. Some studies have shown a significant statistical correlation between *MTHFR* polymorphisms and human male infertility [16], [17], [18], [19], [20], whereas others did not find any such association [21], [22], [23], [24], [25].

In the present study, we investigated the frequency of the C677T and A1298C polymorphisms in the *MTHFR* gene in men with unexplained reduced sperm counts compared with ancestry-matched fertile and/or normozoospermic individuals of Moroccan origin. We observed an association of reduced sperm counts with the *MTHFR* 1298C variant.

## Materials and Methods

### Subjects

We recruited a total of 344 idiopathic infertile Moroccan patients, consisting of 110 men with azoospermia, 89 men with severe oligozoospermia, 58 men with oligozoospermia and 87 men with asthenozoospermia and/or teratozoospermia. All were aged from 25 to 50 years and all underwent an andrological work-up, performed in clinics specializing in male infertility, which included medical history, physical examination, hormonal estimation (FSH, LH, and testosterone) and semen analysis. Patients with known causes of infertility including genetic factors (chromosomal abnormalities and microdeletions in the AZF region of the Y chromosome), lifestyle factors (eg. Smoking, alcoholism, occupation) and clinical factors (varicoele, cryptorchidism) were excluded from the study group. The control group consisted of 450 fertile men who had more than one child and 240 normozoospermic men, all aged from 30 to 55 years. We obtained written informed consent from each subject and the study protocol was approved by the Committee on Research Ethics of Institut Pasteur du Maroc.

### Molecular analysis

Genomic DNA was extracted from peripheral leukocytes using the standard phenol–chloroform method.

**Genotyping of SNP C677T in the MTHFR gene:** The C677T (rs1801133) mutation was analyzed by polymerase chain reaction (PCR) of genomic DNA using the following primer pairs: 5′-TGAAGGAGAAGGTGTCTGCGGGA-3′ (forward) and 5′-AGGACGGTGCGGTGAGAGTG -3′ (reverse). These primers generate a 198 bp fragment. PCR amplification was carried out in a total volume of 10 µL containing approximately 50 ng of genomic DNA, 200 µmol/L dNTPs, 10 pmol of each primer, 1.5 mmol/L MgCl2, 0.5 U Taq polymerase and 1 µL of 10× PCR buffer. The PCR reaction profile was: predenaturation at 94°C for 5 min followed by denaturation at 94°C for 30 s, annealing at 58°C for 30 s and extension at 72°C for 30 s for 35 cycles, with a final extra extension at 72°C for 7 min. PCR amplicons were digested with restriction enzyme *HinfI*. Then the products of digestion were electrophoresed on a 3% agarose gel stained with ethidium bromide and visualized using ultraviolet illumination. The wild type homozygote (CC), heterozygote (CT) and mutant homozygote (TT) showed one band (198 bp), three bands (198, 175 and 23 bp) and two bands (175 and 23 bp), respectively, because the substitution creates a *HinfI* recognition sequence which digests the 198 bp into 175 and 23 fragments.

**Genotyping of SNP A1298C in the MTHFR gene:** The A1298C (rs1801131) mutation was identified using the following primer pairs: 5′-CTTTGGGGAGCTGAAGGACTACTAC-3′ (forward) and 5′CACTTTGTGACCATTCCGGTTTG-3′ (reverse) using the same PCR conditions used for C677T mutation. The amplified fragment of 163 bp was digested with restriction enzyme *MboII*. The A1298C abolishes an *MboII* restriction site. Digestion of the 163 bp fragment of the 1298AA genotype (normal) gives five fragments of 56, 31, 30, 28 and 18 bp, the 1298CC genotype (mutated) results in four fragments of 84, 31, 30 and 18 bp, whereas the 1298AC genotype (heterozygous) produce six fragments of 84, 56, 31, 30, 28 and 18 bp. The digested fragments were separated in a 3% agarose gel stained with ethidium bromide and visualized using ultraviolet illumination.

### Statistical Analysis

The allelic frequencies were calculated by direct counting the alleles. The observed genotype frequencies for fertile and infertile men were compared with each other using Hardy–Weinberg equation (observed allele frequencies) using the Chi squared test. Comparisons of genotype, allele, combination genotype and haplotype frequencies between control and infertile men were done by Chi square test, all p-values were based upon two-tailed tests and Odds ratios (ORs), 95% confidence intervals (CIs) were carried out, *P* values<0.05 were taken as statistically significant.

## Results

We analyzed *MTHFR* polymorphisms C677T and A1298C in 344 infertile and 450 control men using the PCR-RFLP method. The polymorphism distribution of C677T in the *MTHFR* gene is summarized in Table 1. As shown in the table, the frequencies of heterozygous (CT) and homozygous (TT) in the control group were (41.45%) and (7.68%) respectively, these frequencies showed no statistically significant difference compared to the infertile men (36.34% and 5.81%). The *MTHFR* genotypes distribution for the C677T polymorphism was in Hardy-Weinberg equilibrium in controls and all infertile groups. We also determined a possible association of the combined genotypes (CT+TT) and allele T carrier frequencies, but the difference was not statistically significant. In addition, after classifying the patients into different groups, allelic frequencies were not statistically significant between patient and control cohorts.

**Table 1:** Distribution of C677T polymorphism in methylenetetrahydrofolate reductase (*MTHFR*) gene.

								Odds Ratio (95%CI)*						p value*					
Genotype	Controls(n = 690)	Azoopermia(1)(n = 110)	SevereOligozoospermia(2)(n = 89)	Oligozoospermia(3)(n = 58)	Total(1+2+3)(n = 257)	Asth and/or Téra(4)(n = 87)	Total (1+2+3+4)(n = 344)	1	2	3	1+2+3	4	1+2+3+4	1	2	3	1+2+3	4	1+2+3+4
	N(%)	N(%)	N(%)	N(%)	N(%)	N(%)	N(%)												
**CC**	351(50.87)	65(59.09)	53(59.55)	34(58.62)	152(59.14)	47(54.02)	199(57.85)												
**CT**	286(41.45)	37(33.64)	30(33.71)	21(36.21)	88(34.24)	37(42.53)	125(36.34)	0.7163(0.4653–1.09)	0.7186(0.4469–1.139)	0.802(0.4529–1.394)	0.7358(0.5444–0.9909)	1.045(0.6619–1.641)	0.8064(0.6167–1.052)	0.1482	0.1991	0.5218	0.0520	0.9385	0.1298
**TT**	53(7.68)	8(7.27)	6(6.74)	3(5.17)	17(6.62)	3(3.45)	20(5.81)	0.9427(0.4079–1.971)	0.869(0.3296–1.985)	0.6559(0.1577–1.953)	0.8515(0.4718–1.481)	0.4296 (0.1042–1.261)	0.7421(0.4279–1.251)	0.9653	0.9184	0.6617	0.6759	0.2234	0.3293
**CT+TT**	339(49.13)	45(40.91)	36(40.45)	24(41.38)	105(40.86)	40(45.98)	145(42.15)	0.7171 (0.4744–1.078)	0.7036(0.4462–1.101)	0.7312(0.4201–1.258)	0.7155(0.5345–0.9558)	0.8813 (0.5611–1.38)	0.7546(0.5808–0.9794)	0.1337	0.1529	0.3185	0.02811	0.6594	0.04007
**C**	988(71.59)	167(75.91)	136(76.40)	89(76.72)	392(76.26)	131(75.29)	523(76.02)												
**T**	392(28.41)	53(24.09)	42(23.60)	27(23.28)	122(23.74)	43(24.71)	165(23.98)	0.8(0.5712–1.109)	0.7785 (0.5357–1.115)	0.7647(0.4824–1.184)	0.7845(0.6192–0.9903)	0.8274(0.5704–1.184)	0.7952(0.6434–0.9807)	0.2134	0.2086	0.2841	0.04833	0.3509	0.03718

CC, wild type homozyote; CT, heterozygote; TT, mutant homozygote; OR, odds ratio; CI, confidence interval.

Asth, Asthenozoospermia;Tera,Teratozoospermia.

*Controls *vs.*(1),(2),(3),(1+2+3),(4),(1+2+3+4).

The genotype and allele frequencies obtained for the *MTHFR* A1298C polymorphism are presented in table 2. We observed that both the control cohort and a combination of control and infertile men showed highly significant Hardy-Weinberg disequilibrium for the *MTHFR* A1298C polymorphism, (p<1×10^−6^ and p<1×10^−4^ respectively; Tables 3 and 4). The *MTHFR* 1298 variant frequencies showed Hardy-Weinberg equilibrium in the infertile group. This suggests that natural selection is acting against the 1298CC genotype. This is supported by the statistically significant difference in frequencies of this variant between the case and control cohorts. Overall the homozygote CC had an elevated frequency in infertile men (4.94%) compared to the control samples (2.46%) Moreover, this genotype (CC) showed highest frequencies in the severe oligozoospermia group (OR = 3.37, 95% CI = 1.27–8.24; p = 0.01431).

**Table 2:** Distribution of A1298C polymorphism in methylenetetrahydrofolate reductase (*MTHFR*) gene.

								Odds Ratio (95%CI)*						p value*					
Genotype	Controls(n = 690)	Azoopermia(1)(n = 110)	SevereOligozoospermia(2)(n = 89)	Oligozoospermia(3)(n = 58)	Total(1+2+3)(n = 257)	Asth and/or Téra(4)(n = 87)	Total(1+2+3+4)(n = 344)	1	2	3	1+2+3	4	1+2+3+4	1	2	3	1+2+3	4	1+2+3+4
	N(%)	N(%)	N(%)	N(%)	N(%)	N(%)	N(%)												
**AA**	370(53.62)	67(60.91)	49(55.06)	38(65.52)	154(59.92)	51(58.62)	205(59.59)												
**AC**	303(43.92)	39(35.45)	33(37.08)	19(32.76)	91(35.41)	31(35.63)	122(35.47)	0.7019(0.4586–1.064)	0.7529(0.4735–1.184)	0.6226(0.3462–1.092)	0.7004(0.5195–0.9412)	0.7073 (0.4407–1.121)	0.7021(0.5367–0.9166)	0.1184	0.2674	0.1312	0.02220	0.1755	0.01127
**CC**	17(2.46)	4(3.64)	7(7.86)	1(1.72)	12(4.67)	5(5.75)	17(4.94)	1.493(0.4239–4.29)	3.372(1.27–8.238)	0.6948(0.03236–3.945)	1.937(0.888–4.127)	2.41(0.7763–6.473)	2.057(1.025–4.126)	0.6941	0.01431	0.9259	0.1237	0.1626	0.05481
**AC+CC**	320(46.38)	43(39.09)	40(44.94)	20(34.48)	103(40.08)	36(41.38)	139(40.41)	0.7423(0.4894–1.118)	0.9439(0.603–1.472)	0.6089(0.3415–1.062)	0.7735(0.5774–1.034)	0.8164 (0.5162–1.282)	0.7842(0.6027–1.019)	0.1863	0.8869	0.1074	0.09687	0.4428	0.07943
**A**	1043(75.58)	173(78.64)	131(73.60)	95(81.90)	399(77.63)	133(76.44)	532(77.33)												
**C**	337(24.42)	47(21.36)	47(26.40)	21(18.10)	115(22.37)	41(23.56)	156(22.67)	0.8409(0.591–1.181)	1.11(0.773–1.577)	0.6843(0.4113–1.101)	0.8921(0.6994–1.133)	0.9541 (0.6526–1.375)	0.9076(0.7298–1.126)	0.3676	0.6272	0.1562	0.3851	0.8772	0.4104

AA, wild type homozyote; AC, heterozygote; CC, mutant homozygote; OR, odds ratio; CI, confidence interval.

Asth, Asthenozoospermia;Tera,Teratozoospermia.

*Controls *vs.*(1),(2),(3),(1+2+3),(4),(1+2+3+4).

**Table 3:** Hardy-Weinberg distribution for *MTHFR* 1298 genotypes in infertile and control populations.

	Allele X	Allele Y	Observed frequency	%	HW Expected frequency	%	Chi^2^	*P* value
AA	1150	0	575	55.61	600	58.00	1.023	
AC	425	425	425	41.10	375	36.31	6.533	
CC	0	68	34	3.29	59	5.68	10.436	
Total	1575	493	1034	100.00	1034	100.00	17.992	0.000022*

**Table 4:** Hardy-Weinberg distribution for *MTHFR* 1298 genotypes in control population.

	Allele X	Allele Y	Observed frequency	%	HW expected frequency	%	Chi^2^	*P* value
AA	740	0	370	53.62	394	57.12	1.479	
AC	303	303	303	43.91	255	36.91	9.158	
CC	0	34	17	2.46	41	5.96	14.172	
Total	1043	337	690	100.00	690	100.00	24.809	0.00000063*

We also investigated possible associations of different combinations of both polymorphisms between controls and patients (Table 5). The haplotype CCAA showed a slightly significant difference (p = 0.03078) between controls and all infertile patients (OR = 1.389, 95% CI = 1.039–1.855), and the haplotype CCCC showed statistically significant frequencies between the control group and a combination of all patients (OR = 3.313, 95% CI = 1.488–7.659; p = 0.003880), between control and severe oligozoospermia group (OR = 6.687, 95% CI = 2.461–17.72; p = 0.0000449) and between controls and all azoospermia, severe oligozoospermia and oligozoospermia group respectively (OR = 3.326, 95% CI = 1.4–8.035; p = 0.007307).

**Table 5:** Association between the combined *MTHFR* alleles and infertility.

								Odds Ratio (95%CI)*						p value*					
Genotype	Controls(n = 690)	Azoopermia(1)(n = 110)	SevereOligozoospermia(2)(n = 89)	Oligozoospermia(3)(n = 58)	Total(1+2+3)(n = 257)	Asth and/or Téra(4)(n = 87)	Total(1+2+3+4)(n = 344)	1	2	3	1+2+3	4	1+2+3+4	1	2	3	1+2+3	4	1+2+3+4
	N(%)	N(%)	N(%)	N(%)	N(%)	N(%)	N(%)												
**CCAA**	164(23.76)	36(32.73)	22(24.72)	20(34.48)	78(30.35)	26(29.89)	104(30.23)	1.559(1.001–2.402)	1.053(0.6201–1.742)	1.687(0.9394–2.966)	1.397(1.013–1.919)	1.366(0.8254–2.221)	1.389(1.039–1.855)	0.05786	0.9474	0.09672	0.04756	0.2643	0.03078
**CCAC**	186(26.96)	26(23.64)	25(28.09)	13(22.41)	64(24.90)	17(19.54)	81(23.55)	0.8389(0.5164–1.333)	1.058(0.6382–1.719)	0.783(0.3992–1.46)	0.8986(0.644–1.246)	0.6584(0.3681–1.132)	0.8347(0.6162–1.125)	0.5376	0.9206	0.5503	0.5791	0.1758	0.2702
**CCCC**	10(1.45)	3(2.73)	8(8.99)	1(1.72)	12(4.67)	4(4.60)	16(4.65)	1.905(0.4159–6.691)	6.687(2.461–17.72)	1.193(0.0536–7.299)	3.326(1.4–8.035)	3.27(0.8715–10.42)	3.313(1.488–7.659)	0.5629	0.00004489	0.6885	0.007307	0.09839	0.003880
**CTAA**	174(25.22)	24(21.82)	20(22.47)	15(25.86)	59(22.96)	22(25.29)	81(23.55)	0.8278(0.502–1.331)	0.8597(0.4977–1.44)	1.034(0.5457–1.885)	0.8838(0.6275–1.235)	1.004(0.5907–1.662)	0.9134(0.673–1.234)	0.5168	0.6647	0.9611	0.5266	0.9070	0.6095
**CTAC**	113(16.38)	12(10.91)	8(8.99)	6(10.34)	26(10.12)	14(16.09)	40(11.63)	0.6256(0.3191–1.149)	0.5047(0.222–1.032)	0.5895(0.2248–1.337)	0.575(0.36–0.8956)	0.9793(0.5171–1.764)	0.6721(0.4529–0.9842)	0.1853	0.09785	0.3099	0.02048	0.9314	0.05318
**CTCC**	0(0)	1(0.91)	0(0)	0(0)	1(0.39)	1(1.15)	2(0.58)							0.2937			0.6070	0.2186	0.2103
**TTAA**	35(5.07)	7(6.36)	6(6.74)	3(5.1)7	16(6.23)	3(3.45)	19(5.52)	1.271(0.5102–2.836)	1.352(0.5036–3.172)	1.021(0.2421–3.106)	1.242(0.6596–2.266)	0.6687(0.16–2.007)	1.094(0.6054–1.933)	0.7386	0.6807	0.7810	0.5911	0.6905	0.8739
**TTAC**	8(1.16)	1(0.91)	0(0)	0(0)	1(0.39)	0(0)	1(0.29)	0.7823(0.03466–4.959)	0(0.0–4.964)	0(0.0–7.429)	0.3333(0.01483–2.098)	0(0.0–5.072)	0.2488(0.01108–1.564)	0.7983	0.6437	0.8729	0.4778	0.6556	0.2898
**TTCC**	0(0)	0(0)	0(0)	0(0)	0(0)	0(0)	0(0)												

OR, odds ratio; CI, confidence interval.

*Controls *vs.*(1),(2),(3),(1+2+3),(4),(1+2+3+4).

## Discussion

Folates are group B vitamins that play essential roles in the synthesis of nucleic acids and in epigenetic regulation of gene expression through remethylation of homocysteine into methioninine [26]. Changes in folate status could negatively impact on spermatogenesis by causing DNA hypomethylation and thereby disrupting gene expression or by inducing uracil misincorporation during DNA synthesis leading to errors in DNA repair, strand breakage and chromosomal anomalies. There is considerable experimental evidence that key enzymes in the folate metabolism are necessary for male spermatogenesis [19], [24]. Folate deficiency is associated with cardiovascular and obstetrical disorders and neurodegeneration [26], [27], [28]. *MTHFR* gene encodes key regulatory enzyme involved in folate metabolism, and genetic variants of this gene may predispose some men to reduced sperm counts [22], [29]. The C677T variant is associated with a reduction in *MTHFR* activity by 30% in heterozygotes (CT) and 70% in homozygotes (TT) [13]. This polymorphism has been reported to result in mild hyperhomocysteinemia particularly in patients with low folate intake [30]. Our data indicate a lack of association of the C677T variant with infertility. This is in contrast with the reports where the C677T variant was associated with infertility [17], [21], [22], [29], [31]. A study of 77 subfertile men of Dutch ancestory found no significant difference in the frequency of the CC/CT/TT genotypes between the case and the control group, implying a lack of association between for *MTHFR* C677T and infertility, [32]. Another study comprising of 93 infertile and 105 fertile and/or normospermic individuals of Italian ancestry did not find any association between the C677T allele and male infertility [23]. Recently, a meta-analysis was performed in a total of 10 case–control studies, including 2275 cases and 1958 controls; this meta-analysis supports that *MTHFR* C677T polymorphism is capable of causing male infertility susceptibility in Asians, but not in Caucasians [33]. These contradictory results from studies on different populations suggest that the role of C677T in susceptibility to male infertility may depend on environmental factors such as levels of dietary folate uptake [29]. The prevalence of the C677T variant is known to be low (6.6%) in African (Gambia, Kenya, Madagascar and Central African Republic) populations [34]. In Arabs and Berbers Moroccan individuals the frequency of the homozygote TT has been estimated at 6% and the T allele to 26.4% [35]. The frequencies of the genotypyes observed in the current study, TT with a frequency of 5.81% and 7.68% and an T allelelic frequency of 23.98% and 28.41% in the infertile and control groups respectively, supports these reports [34].

The *MTHFR* A1298C variant results in a glutamine to alanine change at codon 429 (exon 7) and is found in a regulatory region of the *MTHFR* enzyme. This variant results in a decrease *MTHFR* activity, which is more pronounced in the homozygous (CC) than in the heterozygous (AC) or wild type (AA) genotype [14]. Although the *MTHFR* p.429A variant is not thermolabile, *in vitro* studies have shown that this variant has approximately 65% of wild-type enzymatic activity compared to that of 40% of wildtype conferred by the C677T variant [13]. Here, we observed a deviation from the Hardy-Weinberg equilibrium for the *MTHFR* 1298 C genotype in the control population. Although a number of factors may be responsible for the observed deviation from the Hardy-Weinberg expectation, we suggest that our data are consistent with purifying selection impacting on the 1298CC genotype. Indeed, we observed a strong association between the 1298CC genotype and severe oligozoospermia (OR = 3.372, 95% CI = 1.27–8.238; p = 0.01431) as compared to the control group. There is growing evidence to indicate that the 1298C variant may be a genetic risk factor for male infertility. A recent study on 151 men with idiopathic infertility and 140 healthy fertile controls of Indian origin concluded that the *MTHFR* 1298CC genotype is a genetic risk factor for idiopathic male infertility in an Indian population [35]. Meta-analyses of the published data concerning MTHFR variants and infertility is inconclusive. A study of 1633 cases and 1735 controls from seven case control studies identified the 1298C allele as a genetic risk factor for infertility, whereas a more comprehensive meta-analyses of 3,850 cases and 4,085 controls from twelve published case–control studies did not observe an association of the 1298C genotype with male infertility, although an association with the *MTHFR* 677T allele was detected [36], [37]. In both of these studies the genetic association was observed in specific phenotypic subgroups and/or in association with specific ethnic groups. Genetic association following phenotype or ethnic stratification was also reported by Wu et al. [33].

In conclusion our data suggest a link between the *MTHFR* variant 1298C and unexplained reduced sperm counts, at least in the Moroccan population. Further studies are necessary to determine the influence of the environment, especially the consumption of dietary folate, on sperm counts of men with different *MTHFR* variants. Since a significant proportion of men with severe oligozoospermia have been reported to have methylation anomalies in their mature sperm [9], [38], the role of *MTHFR* status, dietary folate and methylation profiles and their co-relation in human sperm also merits further investigation.
